# Segmental Congenital Vascular Anomaly With Atrophy, Ulceration and Scarring With Complications in Pregnancy

**DOI:** 10.1111/ajd.14441

**Published:** 2025-02-28

**Authors:** Anne R. Halbert, AmyLeigh Hall, Cathryn Poulton

**Affiliations:** ^1^ Department of Dermatology University of Western Australia Perth Australia; ^2^ Registrar Intensive Care Sir Charles Gairdner Hospital Nedlands Australia; ^3^ Genetic Services of Western Australia King Edward Memorial Hospital Perth Australia

**Keywords:** Horner's syndrome, pregnancy, segmental vascular malformation, sirolimus

## Abstract

A 33‐year‐old woman with a segmental non‐involuting congenital vascular anomaly of the left neck developed pain and ulceration in pregnancy and left‐sided Horner's syndrome after delivery. Mutational analysis of affected tissue showed a missense mutation in *GNA11* (Glu209) and a second mutation in *PIK3CA*. Sirolimus therapy resulted in healing of the ulceration and resolution of the Horner's syndrome.

## 1 Introduction

Segmental congenital vascular anomaly with atrophy, ulceration and scarring (SeCVAUS) is a recently reported entity characterised by a vascular lesion of congenital onset, segmental distribution, red to purple skin colouration with well‐defined geographic margins, variable thickening, central atrophy and episodic ulceration [1]. The entity was first described in the 2022 meeting of the International Society for the Study of Vascular Anomalies (ISSVA) [2], but similar cases have been previously reported as segmental non‐involuting congenital hemangioma [3] and capillary malformation with segmental distribution and central atrophy [4, 5]. Histologically, these lesions are characterised by ectatic small vessels without luminal differentiation in the superficial dermis with ill‐defined solid lobules in the deeper dermis and focally decreased dermal elastic fibres [1, 2]. Immunohistochemical staining of vascular endothelial cells is negative for GLUT1, WT1 and Ki67, with variable D2‐40 positivity [1, 2]. Imaging shows either low flow or low flow admixed with higher flow areas, but no shunting. Genotyping of lesional tissue is available on 13 of 32 published cases [1], revealing postzygotic activating missense mutations in *GNA11*/*GNAQ* (codon 209), as found in congenital hemangioma [6].

## 2 Main Text

**FIGURE 1:**
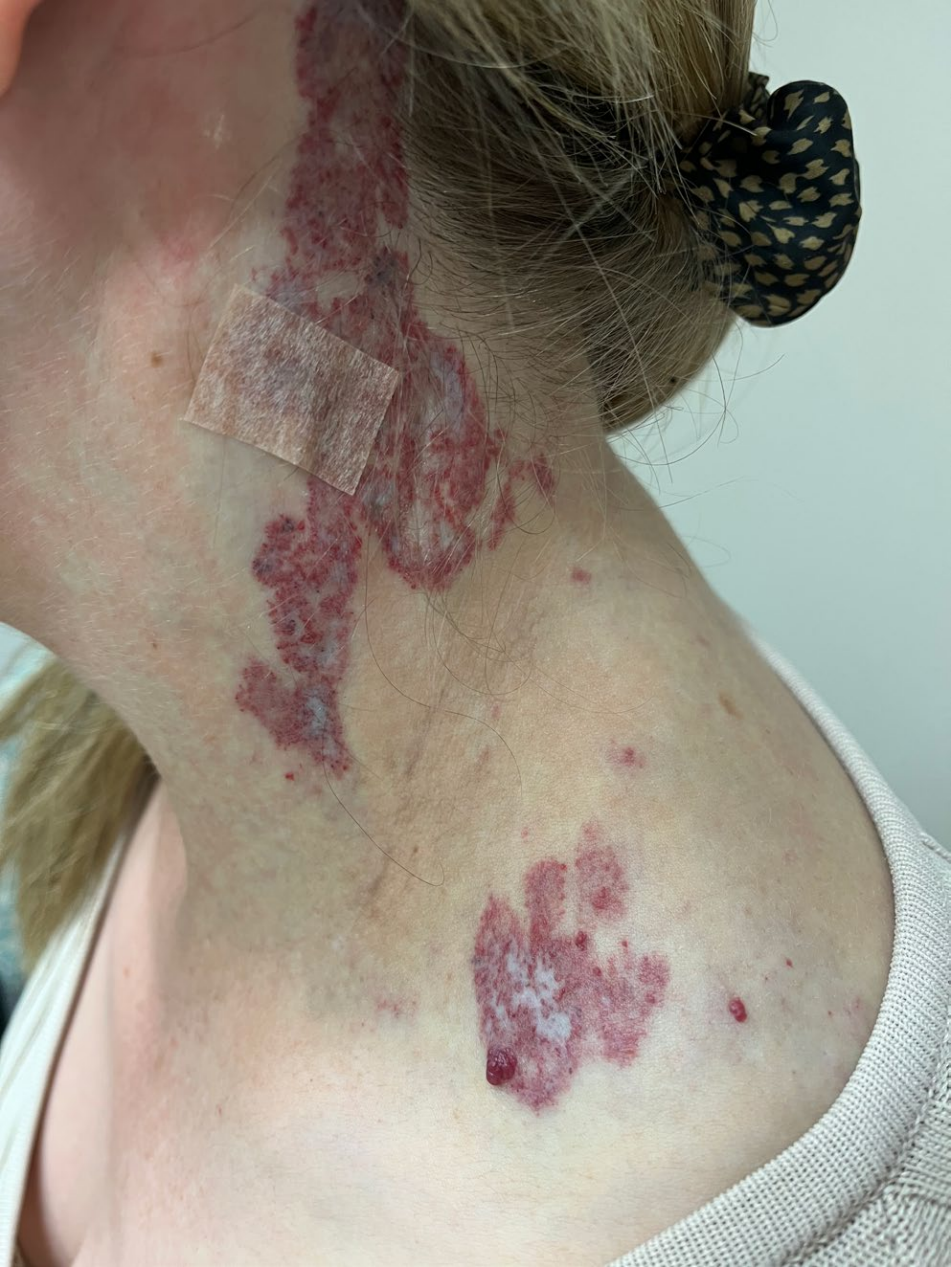
Segmental vascular anomaly with 7 mm area of ulceration beneath the tape and several punctate areas of epidermal necrosis.

**FIGURE 2:**
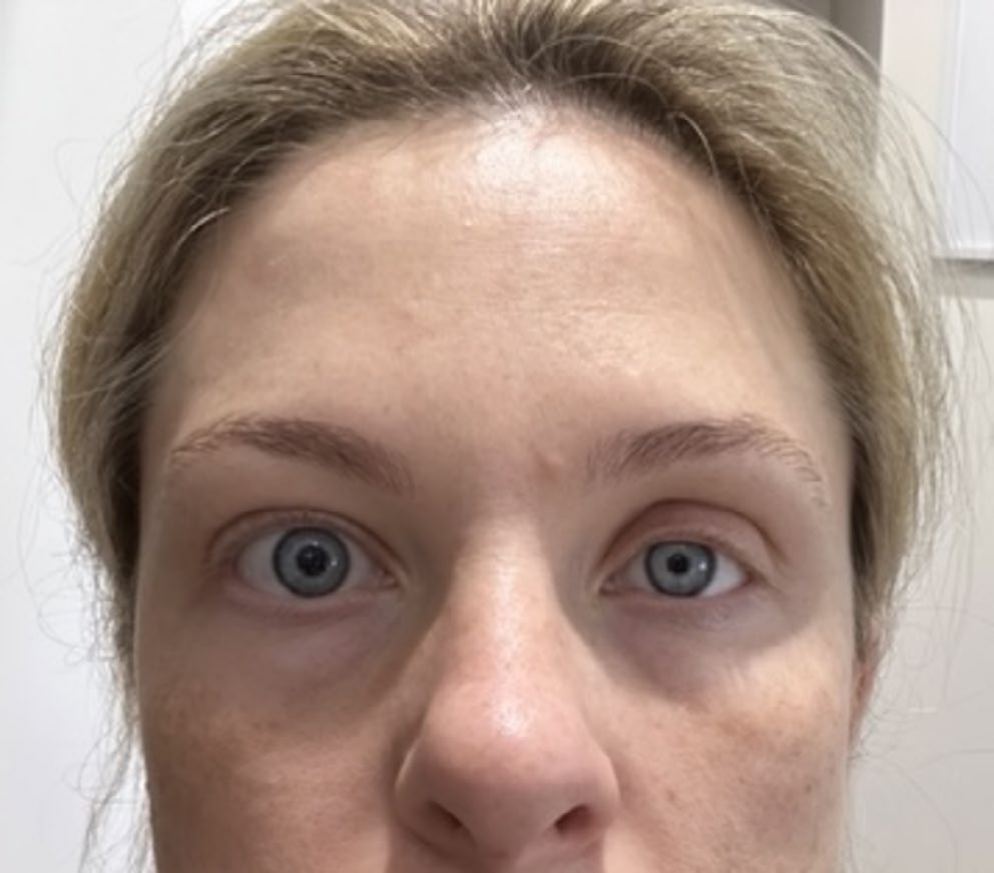
Left sided ptosis and miosis, indicative of Horner's syndrome.

**FIGURE 3:**
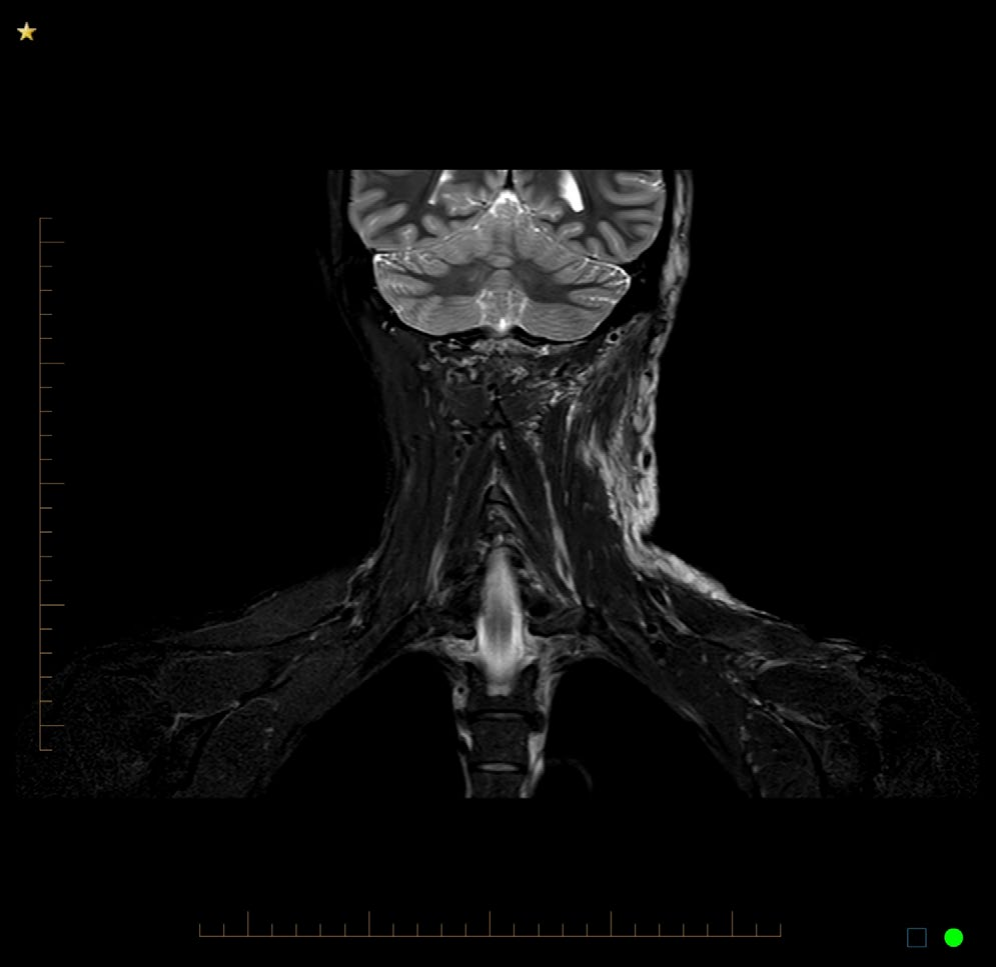
MRI showing T2 hyperintense lesion, more extensive than clinically evident.

A 33‐year‐old woman presented with a painful, ulcerated vascular plaque affecting the left temporo‐occipital scalp, neck and anterior shoulder. The plaque had been present since birth, fading slightly over the years. However, in the second trimester of her first pregnancy, it became more prominent with the development of small papules and nodules (Figure 1) followed by small areas of painful ulceration. Approximately 24 h after the vaginal delivery of her daughter, she developed left‐sided Horner's syndrome with ptosis of the left upper eyelid and miosis of the left pupil (Figure 2). An MRI scan showed an extensive T2‐weighted hyperintense lesion involving subcutaneous tissues of the left neck, root of the neck, infraclavicular fossa, scalp, posterior paraspinal compartment and paraspinal muscles (Figure 3). There was enlargement of several arteries extending to the malformation and prominence of draining veins, but no evidence of shunting. A superficial biopsy showed small, telangiectatic vessels lined by bland endothelial cells, GLUT‐1 and D2‐40 negative. Deep sequencing using a targeted gene panel (Illumina AmpliSeq) on DNA extracted from affected tissue showed a pathogenic variant in *GNA11* c.627G>T p.(Gln209His) at a variant allele frequency (VAF) of 11.8%, supporting the clinical diagnosis of SeCVAUS. In addition, a pathogenic variant in *PIK3CA* c.1633G>A p.(Glu545Lys) at a VAF of 13.8% was isolated.

The patient breastfed her baby for 6 weeks, but with no postpartum improvement in the ulceration and persistence of the Horner's syndrome, she weaned and commenced oral sirolimus 2 mg/d, with trough levels between 6 and 9.6 ng/dL. Within 3 weeks, she felt overall improvement, with less pain, less bulk in the lesion, healing of ulceration and reduced L sided ptosis and miosis. The sirolimus was phased out after 9 months, by which time there was complete resolution of the Horner's syndrome and less bulk demonstrable on repeat MRI. Post cessation, she developed subtle miosis of the left pupil during stressful periods and some increased bulk in the left neck, but no pain or ulceration.

## 3 Conclusion

SeCVAUS is a clinically distinctive lesion with features of both vascular tumour and malformation. It is more common in females (21 of 32 reported cases [1]) and about one third of reported cases have involved the temporo‐occipital scalp and posterior neck [1], as seen in this case. Ulceration in pregnancy was noted in one previously reported patient [1], but our case is notable not only for ulceration but for significant proliferation in pregnancy, followed by the onset of Horner's syndrome after delivery due to involvement of the stellate ganglion. Mutational analysis showed a missense mutation in *GNA11* (209), but this is the first report to show a second mutation in *PIK3CA*. Sirolimus was commenced before the results from mutational analysis became available and coincided with clinical improvement. It is possible the improvement was spontaneous, but the patient felt markedly better within 3 weeks, with all ulceration healing, less pain and less bulk. Sirolimus has been previously tried without success in 3 other published cases of SeCVAUS^1^, so the clinical improvement in our patient may be due to the co‐existing *PIK3CA* mutation. However, of note, sirolimus has been previously reported to be therapeutically useful in the setting of complicated congenital hemangioma [7], also caused by mutations in *GNAQ/GNA11* (209).

In summary, we present a case of newly reported SeCVAUS, which showed progression and ulceration in pregnancy, with enough bulk around the stellate ganglion to cause Horner's syndrome. Improvement was noted after the commencement of sirolimus, which may be due to the *PIK3CA* mutation that was found in addition to the expected *GNA11* (codon 209) mutation.

## Conflicts of Interest

The authors declare no conflicts of interest.

## Data Availability

The data that support the findings of this study are available on request from the corresponding author. The data are not publicly available due to privacy or ethical restrictions.

## References

[1] M. Ivars , I. J. Frieden , L. Provini , et al., “Segmental Congenital Vascular Anomaly With Atrophy, Ulceration, and Scarring (SeCVAUS): Case Series and Review of Literature,” Pediatric Dermatology 41, no. 6 (2024): 1063–1076, 10.1111/pde.15724. 39161100

[2] M. Ivars , I. J. Frieden , L. Provini , et al., “Segmental Non‐Involuting Congenital Vascular Anomaly With Atrophy, Ulceration and Scarring (SNICVAUS): Further Evolution of the Spectrum of “Congenital Hemangioma”. ISSVA World Congress 2022: The Latest in Vascular Anomalies,” Journal of Vascular Anomalies 4, no. 2S (2023): S1–S22, 10.1097/JOVA.0000000000000066.

[3] E. S. West , K. Totoraitis , B. Yadav , et al., “Atypical Presentations of Congenital Hemangiomas: Extending the Clinical Phenotype,” Pediatric Dermatology 36, no. 6 (2019): 835–842, 10.1111/pde.13930. 31576603

[4] M. Ivars , J. M. Azaña , L. Weibel , et al., “Capillary Malformation With Segmental Distribution and Central Atrophy: A Series of 7 Cases,” Journal of the American Academy of Dermatology 83, no. 1 (2020): 213–214, 10.1016/j.jaad.2019.09.016. 31541744

[5] M. P. Rollan , X. Fajre , C. Giordano , C. Whittle , and A. Castro , “Multifocal Capillary Malformation With Segmental Distribution and Central Atrophy: A Case in a 12 Year Old Girl,” Pediatric Dermatology 38, no. 4 (2021): 964–966, 10.1111/pde.14622. 34152623

[6] U. M. Ayturk , J. A. Couto , S. Hann , et al., “Somatic Activating Mutations in GNAQ and GNA11 Are Associated With Congenital Hemangioma,” American Journal of Human Genetics 98, no. 4 (2016): 789–795, 10.1016/j.ajhg.2016.03.009. 27058448 PMC4833432

[7] S. J. Moon , H. J. Baek , B. R. Kim , et al., “Successful Management of Massive Congenital Hepatic Hemangioma and Systemic Hypertension With Sirolimus,” Journal of Pediatric Hematology/Oncology 44920 (2022): e424–e427, 10.1097/MPH.0000000000002146. 33735153

